# Hybrid strategy of graphene/carbon nanotube hierarchical networks for highly sensitive, flexible wearable strain sensors

**DOI:** 10.1038/s41598-021-00307-5

**Published:** 2021-10-25

**Authors:** Yiyi Li, Qinqin Ai, Linna Mao, Junxiong Guo, Tianxun Gong, Yuan Lin, Guitai Wu, Wen Huang, Xiaosheng Zhang

**Affiliations:** 1 Yangtze Delta Region Institute (Huzhou), University of Electronic Science and Technology of China, Huzhou, 313001 China; 2 State Key Laboratory of Electronic Thin Films and Integrated Devices, University of Electronic Science and Technology of China, Chengdu, 610054 China; 3 School of Electronic Information and Electrical Engineering, Chengdu University, Chengdu, 610106 China; 4 School of Electrical Engineering and Automation, Jiangxi University of Science and Technology, Ganzhou, 341000 China

**Keywords:** Condensed-matter physics, Materials for devices, Soft materials, Structural materials

## Abstract

One-dimensional and two-dimensional materials are widely used to compose the conductive network atop soft substrate to form flexible strain sensors for several wearable electronic applications. However, limited contact area and layer misplacement hinder the rapid development of flexible strain sensors based on 1D or 2D materials. To overcome these drawbacks above, we proposed a hybrid strategy by combining 1D carbon nanotubes (CNTs) and 2D graphene nanoplatelets (GNPs), and the developed strain sensor based on CNT-GNP hierarchical networks showed remarkable sensitivity and tenability. The strain sensor can be stretched in excess of 50% of its original length, showing high sensitivity (gauge factor 197 at 10% strain) and tenability (recoverable after 50% strain) due to the enhanced resistive behavior upon stretching. Moreover, the GNP-CNT hybrid thin film shows highly reproducible response for more than 1000 loading cycles, exhibiting long-term durability, which could be attributed to the GNPs conductive networks significantly strengthened by the hybridization with CNTs. Human activities such as finger bending and throat swallowing were monitored by the GNP-CNT thin film strain sensor, indicating that the stretchable sensor could lead to promising applications in wearable devices for human motion monitoring.

## Introduction

Flexible electronics has become a hot area of research attracting extensive attention in recent years^1–4^. Flexible tactile sensor, as a significant subpart of wearable electronics, is usually mounted on wearable equipment to detect human body movement^5–9^. Specially, strain sensors with high sensitivity, stretchability, durability, and rapid response/recovery are essential in monitoring human motion^10^. However, traditional strain sensors based on metal foils or semiconductor films possess unsatisfying flexibility and poor stretchability (ε < 5%) due to the brittleness of sensing materials^11–13^. Therefore, these sensors are not suitable for occasions where both high sensitivity and large stretchable range are required. In order to fabricate high-performance strain sensors, researchers have made tremendous efforts by utilizing various kinds of conductive materials such as silver nanowires^7^, carbon nanotubes (CNTs)^14–16^, CVD graphene^17^, and reduced graphene oxide^18,19^, as the sensing elements.

Networks of overlapping graphene nanoplatelets (GNPs) have been reportedly used in piezoresistive strain sensors, owing to superior stretchability, chemical stability, and economic/scalable synthesis^10,19^. Each GNP is few-layer graphene with thickness of few nanometers. When these platelets connect with each other to form a thin film, the resistance could be influenced by the contact resistances between GNPs. Upon mechanical loading, the change in film resistance originates from the disconnection, cracking, and tunneling effect between GNPs in the film plane^10^. To date, a large variety of flexible strain sensors with large workable strain range and/or high sensitivity have been reported. For example, Wang et al. reported a strain sensor based on buckled graphene film deposited on polydimethylsiloxane (PDMS). The sensor was able to be stretched up to ~ 30%, but showing a small GF of ∼2^20^. Graphene-based strain sensor has been reported with a high gauge factor (GF) of 1000 in the strain range of 2–6%^17^. However, the graphene conductive network could be irreversibly broken, when the applied strain is larger than 7%, leading to the destruction of sensors under deformation. For better practical applications, it’s a significant task to obtain both large workable strain range and high sensitivity. In addition, the fabrication processes of nanomaterials and the strain sensors are generally complicated and costly, possibly generating by-products hazardous to environment. Cost-effective, scalable, and biocompatible approaches are highly demanded for fabricating strain sensors with high sensitivity and large workable strain range.

In this work, we present a strain sensor based on hybridized film of GNPs and CNT, prepared by spray-coating method. CNTs joined GNPs by the *van der Waals* force to form entangled networks. For comparison, strain sensors based on sole GNPs and sole CNTs thin films were also prepared by similar method. The characteristics of thin films and the sensing performance of proposed strain sensors were investigated. Besides, the application of GNP-CNT hybrid thin film strain sensor as a wearable device was demonstrated by mounting it on human finger and front neck to monitor body-motion. It is revealed that CNTs hybridization greatly improves the sensitivity of GNPs and the proposed strain sensor has a promising perspective in applications of human body monitoring.

## Results and discussion

### Fabrication process and device structure

**Figure 1:**
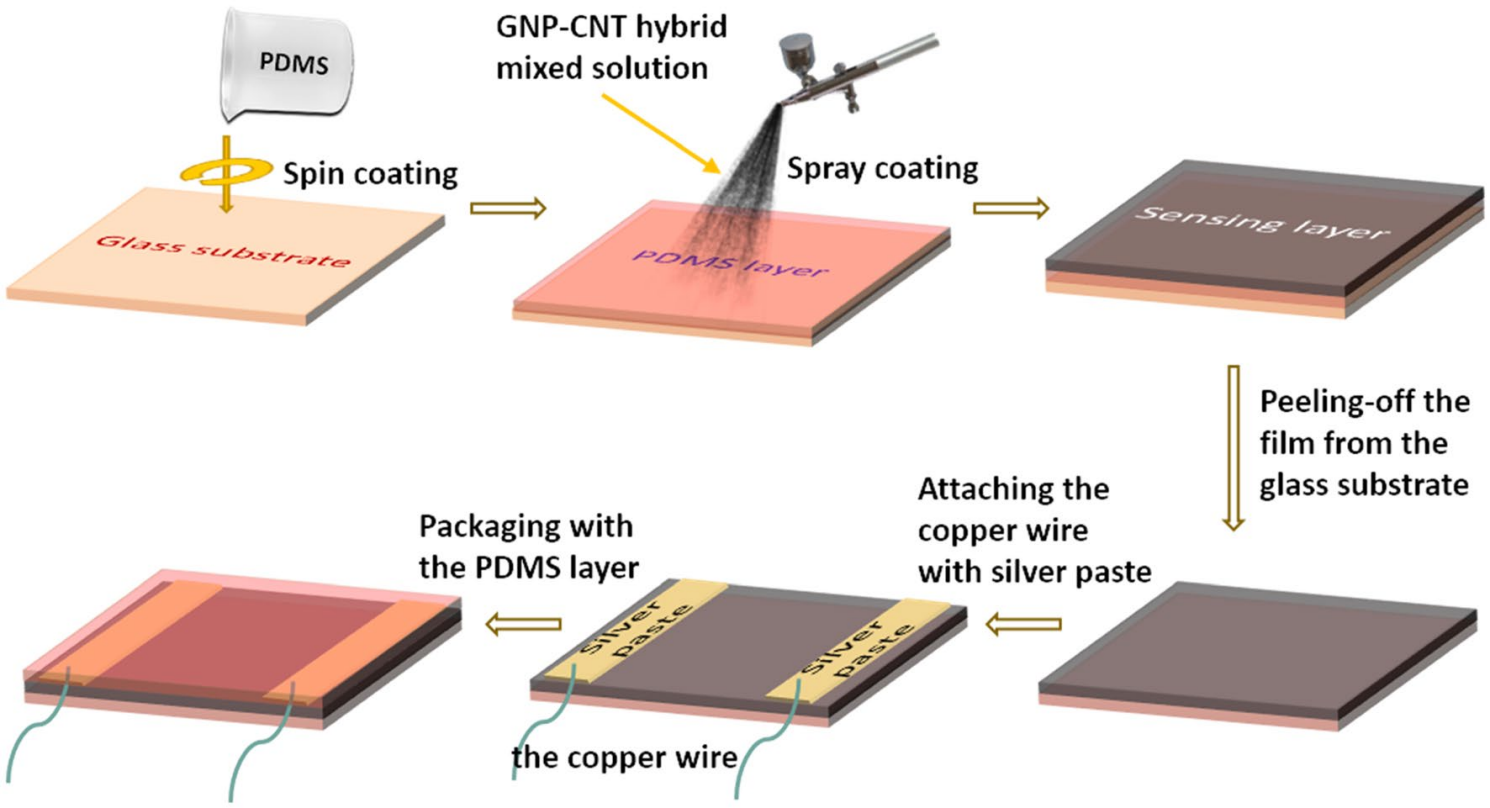
Schematic showing the key steps of fabrication process for the GNP-CNT hybrid thin film based flexible strain sensor by spray coating method. By monitoring the resistance change, tensile strain on the film plane can be detected.

The strain sensors were prepared via a simple spray-coating process. Figure 1 shows schematically the solution-based fabrication of the GNP-CNT hybrid thin film strain sensor. To prepare the hybrid thin films, the GNP-CNT mixed dispersion was made, following the procedure detailed in “Experimental methods” section. Using the sonicated GNP-CNT mixed solution, a spray coating technique was carried out to fabricate the GNP-CNT hybrid thin films. In brief, the GNP-CNT mixed solution was first deposited onto a PDMS substrate at an elevated temperature (controlled at 90 °C) to facilitate solution evaporation. After the spray-coating process, the GNP-CNT/PDMS film was peeled off from the glass slide substrate. Then, silver paste was applied on the two ends of the film to form the electrodes. Finally, the device was covered by a layer of PDMS for encapsulation. Similar procedure was carried out to fabricate strain sensors based on sole GNPs and sole CNTs thin films.

**Figure 2:**
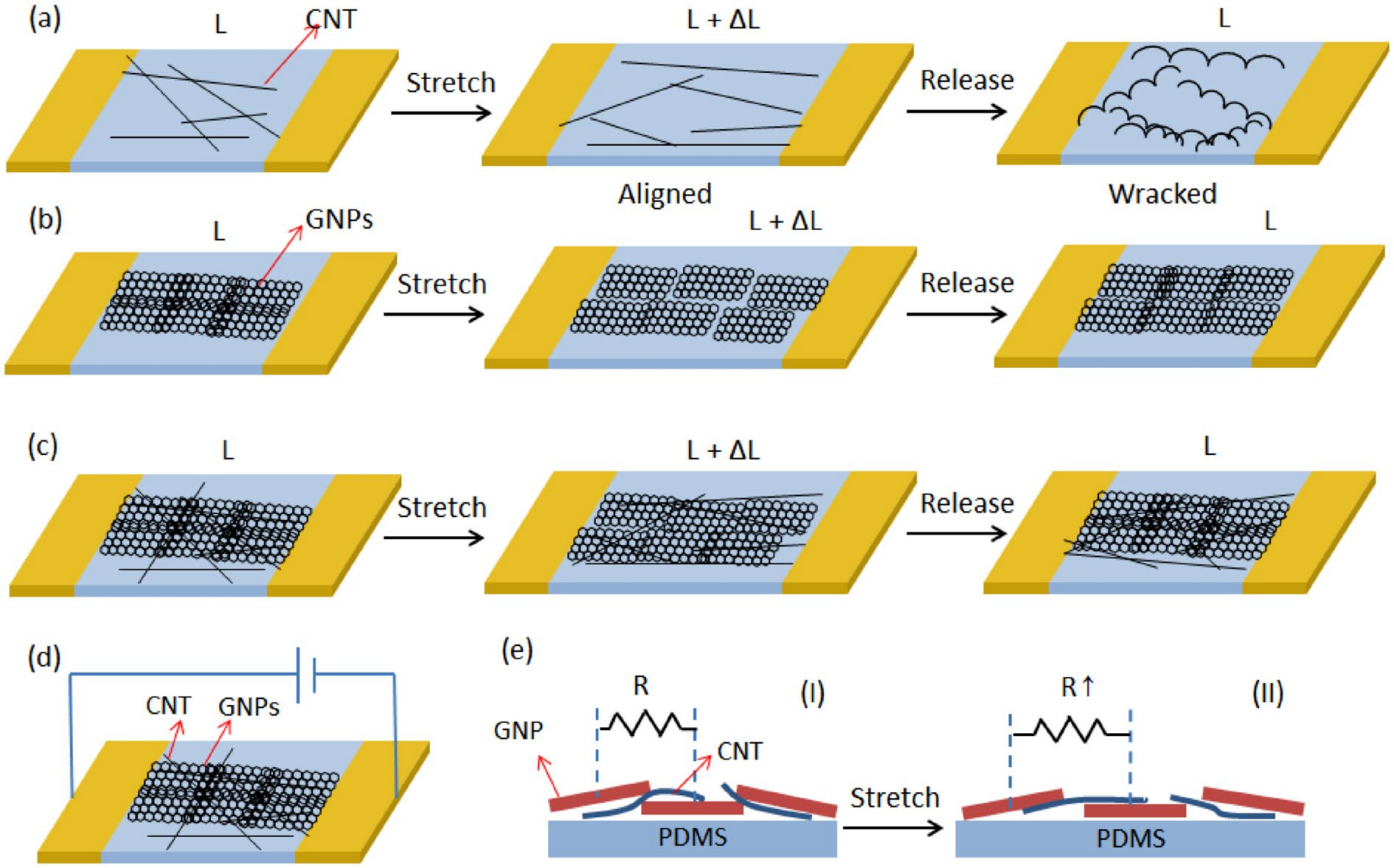
Mechanism of CNTs-enhanced mechanical properties in GNPs network in comparison with the respond of pristine CNTs and pristine GNPs network to repeated mechanical load. Schematic illustration of the microstructural evolution of CNTs (**a**) and GNPs (**b**) and GNP-CNT hybrid (**c**) under stretch and release. (**d**) Measurement scheme of strain sensor based on GNP-CNT hybrid. (**e**) Cross-section of GNP-CNT hybrid sensor, showing stacking GNPs and CNPs before (**e**_**I**_) and after (**e**_**II**_) stretching. Resistance between conductive elements increases after stretching as the disconnections of GNPs and CNTs increase.

Figure 2 shows the working mechanism of three types of strain sensors. Neat CNTs networks are easily destroyed under repeated mechanical load. CNTs bend and buckle upon the release of loads due to the flexibility of 1D nanostructure, which results in wavy structures in the network (Fig. 2a). The generation of nanotube buckles prevents the restoration of conductive paths in the network after releasing of the strain, leading to permanent loss in network conductivity^21,22^. GNPs are loosely connected with each other forming some microcracks and micropores in the 2D nanostructure (Fig. 2b). Under plane strain, GNPs have chances to arrange themselves to maintain connection by reducing overlapped area. Therefore, the connection resistance of GNPs does not change obviously under strain and the GF value is relatively small^10^. Compared with the pristine CNTs network and pristine GNPs network, a GNP-CNT hybrid network is mechanically stronger and more flexible to respond plane strain (Fig. 2c). GNPs are stacking in the dispersion network formed by entangled CNTs, the hybrid network demonstrating properties of both 1D and 2D nanostructure. Due to the weak interactions at the nanotube joints, the networks respond to tensile loads through interfacial sliding between the neighboring nanotubes^23^. Under the same tensile strain, the CNTs show much smaller deformation (slide instead of bending and buckling) than the pristine CNTs network, demonstrating an enhanced strength and better stability. Besides, the flexibility improves because the resistance change of strain sensor based on the GNP-CNT hybrid thin film is not only due to the fracture or crack propagation of GNPs. Figure 2d shows the measurement scheme of a strain sensor based on GNP-CNT hybrid. As demonstrated in Fig. 2e, from relaxed state (Fig. 2e_I_) to tensile state (Fig. 2e_II_), GNPs and CNTs slide by the deformation of the PDMS matrix so that the number of disconnection gradually increases by higher tensile strain, causing the resistance of the strain sensor to increase^11^.

### Characterization

**Figure 3:**
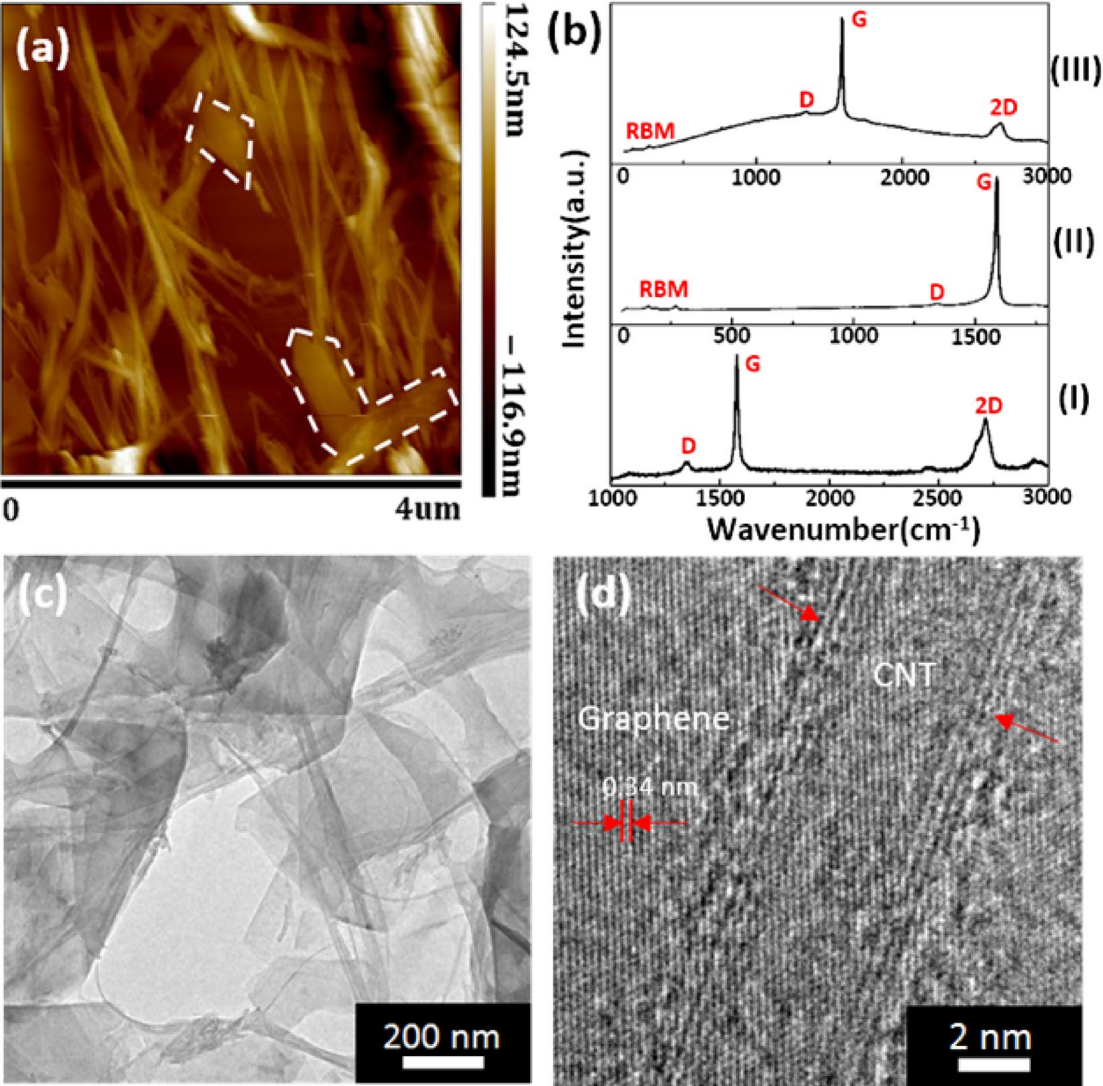
(**a**) Tapping mode AFM images of the GNP-CNT hybrid acquired in a liquid cell. (**b**) Raman image of multi-layer graphene (**b**_**I**_), single-walled carbon nanotubes (**b**_**II**_), and GNP-CNT hybrid materials (**b**_**III**_), respectively. (**c**) and (**d**) TEM and HRTEM images of the GNP-CNT hybrid, respectively. The whiter boxes in (**a**) mark the locations of multi-layer graphene.

The morphology and structure of CNT, few-layer graphene, and the GNP-CNT hybrid material were measured using AFM, TEM, and Raman spectroscopy to demonstrate the percolation theory in thin film. The GNP-CNT hybrid material was fabricated with 5 ml GNPs dispersion and 2 ml CNTs dispersion. Figure 3a shows the AFM image of the GNP-CNT hybrid material acquired in a liquid cell. The whiter boxes in Fig. 3a mark the locations of large GNPs. With AFM imaging, we estimated the lateral dimension (50–2500 nm) and the thickness (~ 4.5 nm) of GNPs. The average bundle length and diameter of the CNTs in GNP-CNT hybrid thin films were determined to be 2000 nm and 5 nm, respectively. It can be seen that the CNTs are intertwined with each other in a mixed structure, while GNPs stacking in the hybrid dispersion network. Raman spectrum of few-layer graphene, CNT, and GNP-CNT hybrid materials are presented in Fig. 3b. Figure 3b_I_ presents a strong G band at 1578 cm^−1^ but rather weak D band at 1349 cm^−1^, suggesting that the few-layer graphene is mainly constituted of sp^2^ hybridized carbon^24^. The 2D band (2714 cm^−1^ in Fig. 3b_I_) of few-layer graphene has been regarded as a sensitive indication of the number of layers^25–29^. The radial breathing mode (RBM) as well as the G and D band on the spectrum (Fig. 3b_II_) is markers for determining the diameters of the CNT^30,31^. Focusing on the RBM of the spectrum, the Raman shift at 68.6 cm^−1^, 157.7 cm^−1^, 191.2 cm^−1^ and 270.5 cm^−1^ respectively correspond to CNTs approximately 3.98 nm, 1.54 nm, 1.25 nm and 0.87 nm in diameter^32^. Raman spectroscopy unambiguously corroborates the presence of GNP-CNT hybrid material as shown in Fig. 3b_III_. The connections between GNPs and CNTs are important for achieving hybrid materials with good performance. Microstructures and morphology of the GNP-CNT hybrid material imaged at different magnifications were investigated by TEM. TEM and HRTEM images of GNP-CNT hybrid material are shown in Fig. 3c,d, respectively. From the TEM image, the junctions between GNPs and CNTs are clearly observed. The HRTEM image (Fig. 3d) confirms that small lattice fringes with an interlayer thickness of 0.34 nm, which belongs to sp2 bonded graphite carbon. The diameter of the carbon nanotubes is about 5 nm, which is consistent with the AFM result. Both images clearly reveal that GNPs and CNTs are uniformly dispersed to form the mixed structures of CNTs entangled network stacking by GPNs in the hybrid dispersion.

**Figure 4:**
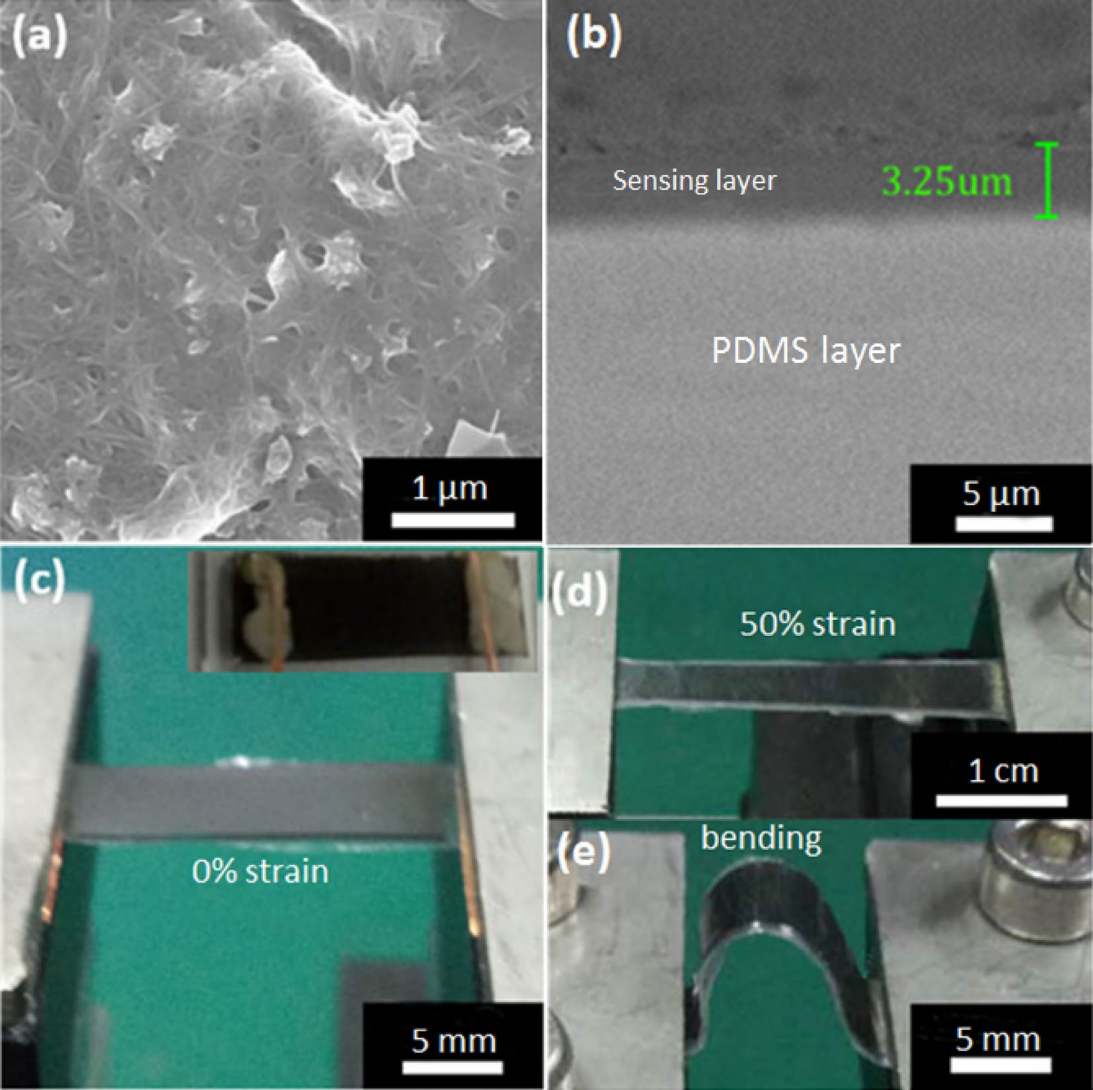
(**a**) Photograph of the GNP-CNT hybrid thin film strain sensor. (**b**) SEM image of GNP-CNT hybrid thin film, showing the cross-section view. (**c**–**e**) Photographs of a GNP-CNT hybrid thin film strain sensor at its original length and 50% of stretching and bending of strain sensor, showing the superb stretchability of the sensor.

The SEM images of surface morphology and the cross-section of the GNP-CNT hybrid thin film were shown in Fig. 4a,b, respectively. CNTs are uniformly dispersed within the network of percolating GNPs as seen in Fig. 4a. The thickness of the hybrid thin film is estimated to be ~ 3.25 μm from Fig. 4b. From the SEM results, it suggests that the CNTs rope entangled network structures stacking mosaic morphology of GNPs in the as-prepared thin film. Figure 4c,d are photographs of a strain sensor made of GNP-CNT hybrid thin film at its original length and with over 50% strain, respectively, showing the stretchability and bendability of the sensor. Figure 4e shows the photographs of the sensors under bending stress with ultra-softness. The sensors can be directly mounted on human skin or attached to complex surfaces with perfect contact and negligible slippage.

### Piezoresistive characteristics analysis

The GNP-CNT hybrid thin film sensor was clamped to a motorized moving controller (WNMC 400 Motion Controller) to characterize its electromechanical behavior. Multiple strain/release cycles with different strain levels were applied to the sensor, while the resistance changes were measured. The gauge factor (GF) was evaluated according to the coupled electrical-cyclic tensile testing results:

1 $$GF=\frac{\Delta R/{R}_{0}}{\Delta L/L}$$

**Figure 5:**
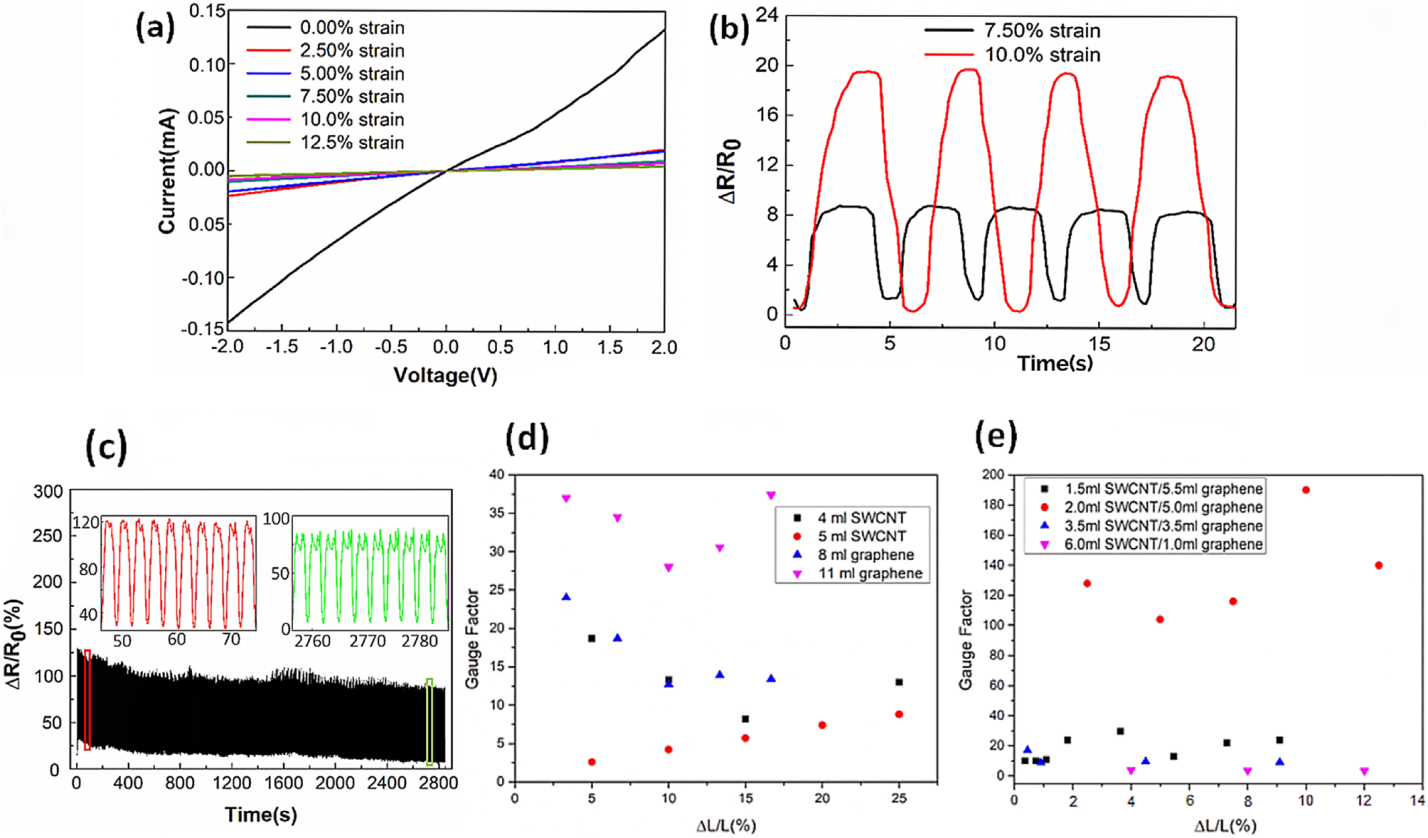
Piezoresistive performance of thin film sensors. (**a**) Measured I-V curves of the GNP-CNT hybrid thin film strain sensor with various applied strain levels. (**b**) The ΔR/R responses of the GNP-CNT hybrid thin film sensor under 7.5% strain and 10% strain. (**c**) Plot of the resistance response over 1000 loading/unloading cycles at a strain of 10%. (**d**) Effect of different strain level on the gauge factors of sole CNTs and sole graphene thin film sensors. (**e**) Effect of different strain level on the gauge factors of GNP-CNT hybrid thin film strain sensor with different GNP/CNTs ratio. The optimal volume ratio of graphene dispersion to CNTs dispersion is 5:2.

$\Delta R$ is the relative change in resistance. ${R}_{0}$ is initial resistance without applying strain. $\Delta L/L$ is the displacement variation of the sensing film. The I–V curves (Fig. 5a) at different strain levels indicate that the response of the sensor based on GNP-CNT hybrid thin film to strain is steady, and the resistance (slope of I–V curves) under each applied strain is constant. The slope of the curve under strain conditions is much smaller than that of the curve when no strain is present, indicating a significant increase in the resistance.

When the strain sensor was held at various levels of strain, the resistance changes were recorded to evaluate the stability of output signal. During each period, the signals remained stable without distinct drifts, as shown in Fig. 5b, the Gauge Factors of strain sensor are 116 under 7.5% strain and 197 under 10% strain, respectively. The GF values, calculated to be 10–197, were superior to those of previously reported stretchable sensors (GF: 0.5–69)^7,33,34^ and some other graphene/CNTs based strain sensors (GF 0.54 at 90% strain, GF 15 at 206% strain)^35,36^.

Our sensor also showed long-time stability, little hysteresis, and high durability. Figure 5c shows the response of the sensor to 1000 times cyclic loading of 10% strain, indicating its remarkable stability. For comparison, strain sensors based on sole GNPs and sole CNTs thin films were prepared and measured by similar method. Figure 5d compares the GF values of sole CNTs thin film sensors (sprayed with 4 mL or 5 mL CNTs dispersion, sensing layer thickness ~ 2.0 μm or ~ 2.1 μm) with those of sole GNPs thin film sensors (sprayed with 8 mL or 11 mL GNPs dispersion, sensing layer thickness ~ 2.9 μm or ~ 3.1 μm). Figure 5e compares the GF values of GNP-CNT hybrid thin films fabricated with altered GNP/CNTs ratio under different strain levels. The sensitivity of strain sensors fabricated with optimal GNP-CNT volume ratio 5:2 is much better than that of sole CNTs and sole GNPs thin films sensors. Besides, consistent with the mechanism described in Fig. 2, after the experimental strain/release cycles, the resistance of strain sensor made of sole CNTs failed reverting to initial value and that of strain sensor made of CNT/GNP or sole GNPs was able to resume to initial value.

**Figure 6:**
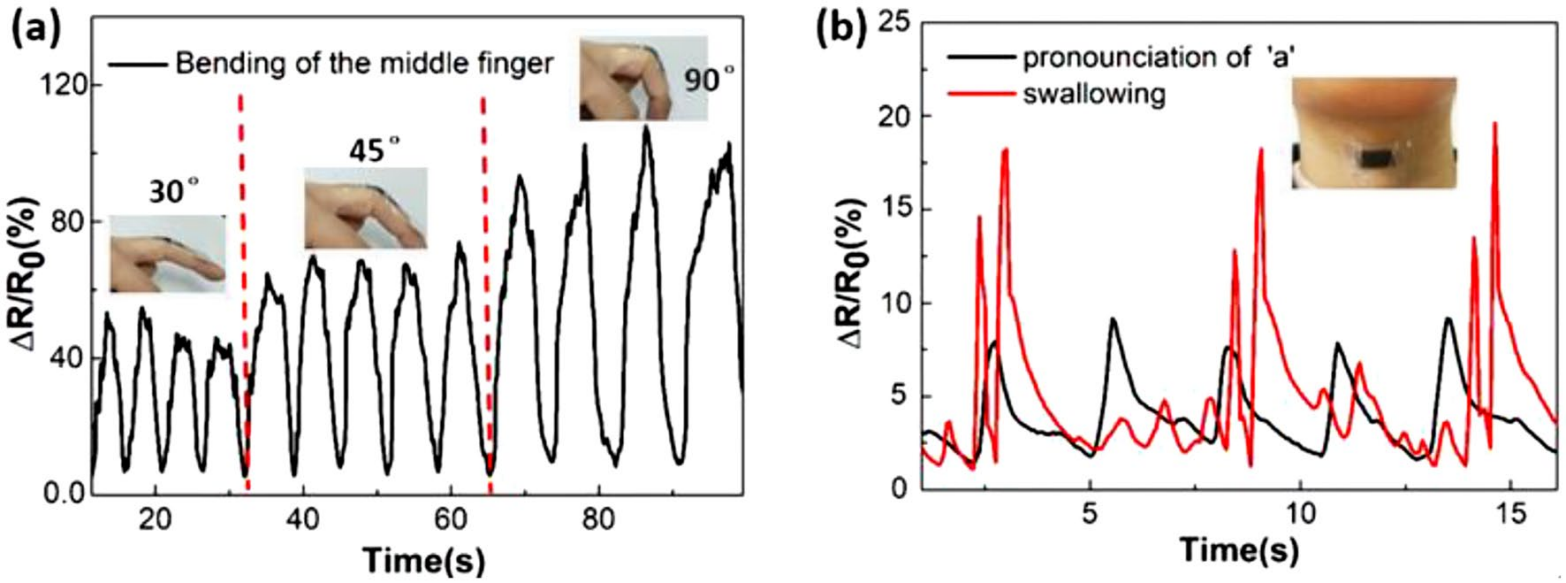
Strain sensor used to monitor human motion. (**a**) Relative change in resistance while bending the strain sensor at joint area of a finger. (**b**) Resistance change in response to pronouncing ‘a’ and swallowing of the throat.

The skin deformation combines stretching and bending^37^, which could lead to the structural changes of these microcracks and micropores^38^, thus the film could be used as a skin-mountable strain sensor to detect human motion. In comparison to the traditional strain sensors based on metal foil or silicon, the unique feature of GNP-CNT hybrid thin film strain sensors is their remarkable extensibility and durability^20,39^. In combination with the high sensitivity and ease of fabrication, these properties make the strain sensor highly promising for applications in human–machine interactions and body motion monitoring. To demonstrate the potential and applicability, the GNP-CNT hybrid thin film strain sensor was mounted on the finger and the front neck to monitor body motion. We fixed the strain sensor on a middle finger to see its resistance response to the bending of the finger (Fig. 6a). When the middle finger slowly bended toward the palm to a certain angle (30°, 45°, and 90°) and subsequently released repeatedly, the finger motion was faithfully registered by the continuous increase and decrease of the resistance. The greater the bending angle is, the more the resistance increases. The resistance almost doubles when the middle finger bends to an angle of 90°. By mounting the strain sensor on human finger, the GNP-CNT hybrid thin film strain sensors could potentially be useful for a broad range of applications in human–machine interactions, such as making phone calls, clicking the mouse, typing on the keyboard, playing piano and remotely controlling the operation of vehicles. Moreover, the strain sensors based on GNP-CNT hybrid thin film can be used for body monitoring. Figure 6b shows a GNP-CNT hybrid thin film strain sensor attached onto skin of the neck to noninvasively monitor the muscle movement during pronouncing ‘a’ and swallowing. The strains induced by the motion of surficial skin along a human neck were clearly sensible with reproducible signals in resistance. The GNP-CNT hybrid thin film strain sensor exhibited high sensitivity and distinct current curves while pronouncing ‘a’ or swallowing. This further demonstrated the excellent performance of the strain sensor in voice recognition.

## Conclusion

In summary, we demonstrate a simple, cost-effective, and scalable approach for the implementation of highly sensitive and stretchable strain sensors based on GNP-CNT hybrid thin films. Strain sensors with high sensitivity (GF ~ 197 under 10% strain), high stretchability (ε ≥ 50%) and a reproducible response over 1000 loading cycles (including stretching and bending) can be achieved by incorporating CNTs into GNPs networks to form 1D-2D hybrid microstructure. The devices can be used for strain and vibration sensing in a variety of applications ranging from human physiological activity monitoring to soft robotics.

## Experimental methods

### Ethical approval

The study was approved by the Ethics Committee of University of Electronic Science and Technology of China. All procedures performed in this study involving human participants were carried out in compliance with the Declaration of Helsinki. The informed consent was obtained from all the human participants in the study.

### Materials and chemicals

Graphite powder (≥ 325 mesh, 99.95% metals basis) was purchased from Aladdin Biochemical Technology Co. Ltd. TNWDIS (90 wt%) and single-walled carbon nanotubes with an average length of 5–30 μm and average diameter of 1–2 nm were purchased from Chengdu Organic Chemicals Co. Ltd, Chinese Academy of Sciences. N-Methylpyrrolidone (NMP, 99.13 (MW), analytical pure) was purchased from Kelong Chemical Reagents Factory. The PDMS monomer and curing agent were provided by Sylgrad-184, Dow Corning. All the materials and chemicals were used as received without further purification.

### Preparation of GNPs dispersion and CNTs dispersion

Graphene dispersion was obtained from exfoliation of natural graphite powder based on Chiang’s liquid exfoliation method^40,41^. Graphite dispersions were prepared by dispersing natural graphite in N-Methylpyrrolidone (with the mass fraction 0.2 of deionized water) at an initial concentration of 5 mg/ml. These dispersions were sonicated in a pulse operation mode (on 2 s, off 3 s) with the power set at 60 W for 15 h, followed by standing for 24 h to allow the formation of any unstable aggregates in the bottom. The dispersion was then centrifuged at a speed of 3000 rpm for 15 min. The supernatant obtained was the graphene dispersion. The CNTs dispersion (0.3 mg/ml) was prepared by sonicating a mixture of 30 mg CNTs and 105 mg TNWDIS in 100 g deionized water. The sonication conditions, except the sonication duration (2.5 h), were the same as those of preparing GNPs dispersion. The dispersion was then centrifuged at a speed of 2000 rpm for 15 min. The supernatant obtained was the CNTs dispersion.

### Fabrication of GNP-CNT hybrid nanomaterials

The GNP-CNT hybrid nanomaterials were prepared by introducing CNTs solution into GNPs dispersion. To make the hybrid mix uniformly, the mixture was sonicated for 2.5 h under stirring with the speed of 1000 rpm/min to form a homogeneous suspension.

### Fabrication of strain sensor

The strain sensor was fabricated by spraying 10 ml diluted GNP-CNT hybrid mixed dispersion (7 ml dispersion with 3 ml DI water) onto an elastomeric polydimethylsiloxane (PDMS) substrate. Spray coating was carried out with a commercial airbrush (ACG model HD-130, Taiwan). In the spray process, the PDMS substrate was heated by a hot plate set at 90 °C to accelerate the solvent evaporation and facilitate thin film formation. The GNP-CNT hybrid coating film was gradually formed by controlling the spraying speed to balance the spraying and solvent evaporation time. For all the thin film sensors, silver paste was applied on the two ends of the film to form a two-electrode configuration for the afterward sensing performance evaluation.

### Characterization

Scanning electron microscopy (SEM) was performed with Hitachi S-4800 at 5.0 kV for examining the morphologies and thicknesses of different sensors. The tapping mode AFM (Veeco Digital Instruments by Bruker Dimension D3100) was used to acquire images of GNP-CNT hybrid nanomaterials deposited on silicon wafer for thickness measurement. Structural and morphological characterization of the material was performed on a FEI Tecnai G2 F20 TEM operated at 200 kV of accelerating voltage. The two ends of the sensor were mounted on a customized micrometer moving stage, and the sensing film can be bent and stretched by moving the stage closer. Electrical properties of the sensor were collected by a source measurement unit (SMU) instrument (Keithley 2400).
